# Inflammatory Response: A Crucial Way for Gut Microbes to Regulate Cardiovascular Diseases

**DOI:** 10.3390/nu15030607

**Published:** 2023-01-24

**Authors:** Wen Wang, Luo-Jiang Zhu, Yue-Qi Leng, Yu-Wan Wang, Te Shi, Wei-Zhong Wang, Jia-Cen Sun

**Affiliations:** 1 Department of Marine Biomedicine and Polar Medicine, Naval Medical Center, Naval Medical University, Shanghai 200433, China; 2 Department of Neurosurgery, 922th Hospital of PLA, Hengyang 421000, China; 3 Department of Gastroenterology, Naval Medical Center, Naval Medical University, Shanghai 200433, China

**Keywords:** gut microbiota, inflammatory response, metabolite, cardiovascular disease, prebiotics and probiotics

## Abstract

Gut microbiota is the largest and most complex microflora in the human body, which plays a crucial role in human health and disease. Over the past 20 years, the bidirectional communication between gut microbiota and extra-intestinal organs has been extensively studied. A better comprehension of the alternative mechanisms for physiological and pathophysiological processes could pave the way for health. Cardiovascular disease (CVD) is one of the most common diseases that seriously threatens human health. Although previous studies have shown that cardiovascular diseases, such as heart failure, hypertension, and coronary atherosclerosis, are closely related to gut microbiota, limited understanding of the complex pathogenesis leads to poor effectiveness of clinical treatment. Dysregulation of inflammation always accounts for the damaged gastrointestinal function and deranged interaction with the cardiovascular system. This review focuses on the characteristics of gut microbiota in CVD and the significance of inflammation regulation during the whole process. In addition, strategies to prevent and treat CVD through proper regulation of gut microbiota and its metabolites are also discussed.

## 1. Introduction

Cardiovascular diseases (CVD) pose great threat to human health. Inflammation is a prevalent cause of the CVD pathological process, including immune cells activation, accumulation, and inflammatory factors release. Recent research reported in the NEJM that canakinumab targeting IL-1β could reduce the risk of cardiovascular events caused by atherosclerosis [1], which indicated the important role of inflammation in atherosclerosis. In addition, improved prognosis was improved in patients with heart failure or coronary vascular disease treated with steroids for its anti-inflammation effect [2]. However, the development of innovative strategies treating cardiovascular diseases remains sluggish. Growing evidence has proven that an indispensable role of gut microbiota in the interaction with the cardiovascular system is through gut–brain axis or gut–heart axis, etc., and that inflammation regulation is believed to be an important part. Therefore, exploring the deeper underlying molecular mechanisms of inflammation in gut microbiota-related cardiovascular diseases is necessary. This review aims to present succinct outlines of gut microbiota involvement and its metabolites in CVD. We also address the importance of inflammatory response from the view of gut microbiota mediated biological reaction, which could offer a new entrance for CVD treatment.

Gut microbiota refers to the microorganisms existing in the intestinal tract of animals, participating in a variety of physiological and biochemical processes, and playing an important role in maintaining stability of the internal environment [3]. The intestinal tract is not only an organ for digestion and absorption, but also the largest immune organ. The functional transformation of gut microbiota has been recognized as potential mediators of intestinal immune functions, including activating immune cells, releasing inflammation factors, and forming an inflammatory microenvironment [4]. Dysbiosis of gut microbiota has been proven to be responsible for the occurrence and development of chronic kidney diseases, atherosclerosis, and heart failure, where exorbitant inflammatory response was an integral part [5,6]. Researchers have found that MBP (Mesona chinensis Benth Polysaccharide) reduced the abundance of *Helicobacter pylori* and *Prevotella*, while increasing the *Lactobacilli* and *Coprococcus*, effectively blunted the overproduction of TNF-α and IL-1β as well as the activity of myeloperoxidase. Additionally, the underlying mechanism was via inhibiting the activation of the TLR4/MAPK/NF-KB pathway [7]. The regulation of inflammatory factors by gut microbes involves serum and organs like kidney and heart [8,9]. Inflammatory bowel disease was highly related to disturbed gut microbiota, while probiotics can mitigate colitis through modulating host immunity and the gut microbiome [10]. Clinical symptoms gained considerable improvement after probiotics administration, accompanied by a healthier change of serum cytokines, including a significant decrease of serum pro-inflammatory factors, Interleukin-6 (IL-6), Interleukin-1β (IL-1β), tumor necrosis factor-α (TNF-α), and a sharp increase of the serum anti-inflammatory factor Interleukin-13 (IL-13) [11]. Evidence showed that Paeonol restricted the development of atherosclerosis with respect to the restoration of the balance of Treg/Th17, which indirectly downregulated the protein expression of LOX and fibrosis-related indicators (MMP-2/9 and collagen I/III). The underlying mechanism was associated with increased microbiota-derived SCFA production [12].

Inflammation is widely involved in the pathogenesis of atherosclerosis, heart failure, myocardial infarction, and hypertension [13]. However, the close relation between gut microbiota and inflammatory response in CVD was sometimes overlooked. The Qige Huxin formula (QHF) has been widely used in clinical heart failure treatment for decades. Recent study has shown that QHF increased the relative abundance of *Marvinbryantia* and *Phascolarctobacterium* and decreased the levels of TNF-α, IL-1β, and IL-6 in serum, exerting numerous cardioprotective effects [14]. Gut microbiota disruption stimulated inflammatory responses through metabolic changes, secretion of inflammatory factors, and intestinal mucosal destruction [15,16]. Therefore, this review focuses on the effects of gut microbiota on cardiovascular diseases from the perspective of inflammation so as to provide new insights into the prevention and treatment of CVD.

## 2. Enterotype and Inflammatory Responses

### 2.1. Enterotype and Intestinal Microenvironment

The earliest research about the genomics of gut microbiota emerged in 2010, and the genome catalogue of human gut microbial colonies was published in *Nature* [17]. A total of 3.3 million effective reference genes of the human gut microbiota genome were obtained, which was about 150 times that of the human genome. The gastrointestinal (GI) tract was estimated to be colonized by at least 1000~1150 species of bacteria, with an average of 160 dominant bacteria [17]. Further studies showed that the human gut microbiota with different ages, weights, genders, and ethnic groups could be roughly divided into three types: *Bacteroides*, *Prevotella*, and *Ruminococcus* [18,19]. They restricted and depended on each other, forming an ecological balance in terms of quality and quantity.

Currently, enterotype is highly associated with diet, nutrition, and metabolism [20]. *Bacteroides* can effectively break down carbohydrates, making it easier to intake energy from food [21]. *Prevotella* tends to degrade mucoprotein and decompose cereals and legumes. *Ruminococcus* contributes to sugar absorption, thus leading to obesity [18,22]. The composition of gut microbiota varies from person to person, leading to a unique microenvironment. In recent years, the concept of the intestinal microenvironment was prevalent and is believed to be composed of intestinal microorganisms, intestinal mucosal epithelial tissues, and immune organ in the intestinal mucosa, taking great part in absorption, metabolism, as well as immune regulation. Among them, gut microbiota were considered the core components [23].

A stable gut microbiota strikes positive effect on maintaining the normal histological and anatomical structure of the organs, promoting vitamin synthesis, sustaining intestinal pH value, promoting ion absorption, and participating in substance metabolism. In addition, gut microbiota also participates in the formation of the intestinal mucosal barrier and interaction with submucosal immune cells to regulate the immune system [24,25]. Thus, the mucosal immune system and the homeostasis of gut microbiota are interdependent, and a balance between them maintains a stable intestinal environment.

### 2.2. Two Ways of Regulating Inflammatory Response

**Figure 1:**
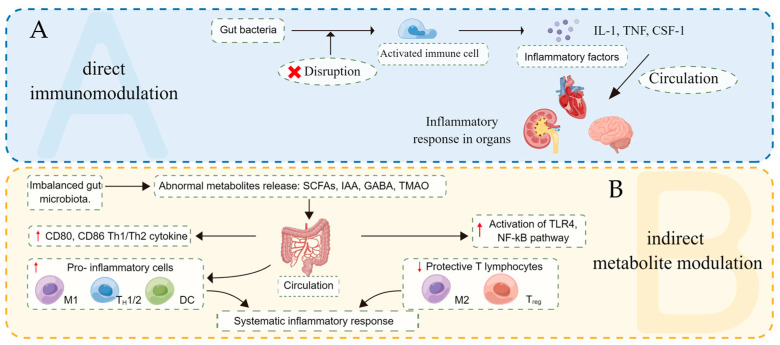
Two ways of regulating inflammatory response through gut microbiota. (**A**) Disrupted gut microbiota stimulate inflammatory response in damaged organs through activating immune cell and releasing pro-inflammatory cytokines. (**B**) Abnormal metabolites released by imbalanced gut microbiota facilitate inflammatory response by transforming the phenotype and function of immune cells and activating the inflammation-related pathway. IL-1: Interleukin 1; TNF: Tumor necrosis factor; CSF-1: Colony stimulating factor 1; SCFAs: Short-chain fatty acids; IAA: indoleacetic acid; GABA: Gamma-aminobutyricacid; TMAO: trimethylamine *N*-oxide; TLR4: Toll-like receptors 4; NF-κB: nuclear factor-κB; M1: type I macrophage; M2: type II macrophage; Th1/2: T helper cells type 1/2; DC: Dendritic cell; Treg: Regulatory T cells. Created with Figdraw.

Currently, there are mainly two common ways for gut microbiota to regulate the inflammatory response (Figure 1). One is being recognized by the immune cells directly and induced innate or adaptive immune responses [26,27], which is confirmed in inflammatory bowel disease [28]. The other is to modify the function of immune cells by producing active substances, such as short-chain fatty acids (SCFAs), indoleacetic acid (IAA), trimethylamine *N*-oxide (TMA-O) [29,30,31,32].

SCFAs are mainly composed of acetate (C_2_), propionate (C_3_), and butyrate (C_4_), which modulate the immune responses not only in the gut but also in distal mucosal sites, such as the lungs, heart, brain, and kidney. In the gut, the formation of microbial SCFAs leads to a reduced pH of the lumen, resulting in the inhibitory growth of *Enteropathogens*. *Bifidobacteria* produce SCFAs, particularly acetate and butyrate [33,34], among which the butyrate has been prominently known for its anti-inflammatory effect [35]. The SCFAs play a pivotal role in maintaining and establishing mucosal immunity. For example, SCFAs were identified to promote goblet cells differentiation, mucus production, and tight junction permeability, thereby enhancing the function of the intestinal epithelial barrier [36]. Moreover, SCFAs were also responsible for sustaining the intestinal homeostasis by stimulating anti-inflammatory actions through the involvement of Regulatory T cells (Treg). Mice lacking Treg cell activity were more prone to the development of intestinal inflammation. Additionally, butyrate and propionate fortified diets in mice were reported to increase the amount of Treg cells in the colon and control the inflammatory response [37].

As the metabolites of the gut microbiota, SCFAs production is highly dependent on the function of gut microbiota. The complexity of gut microbiota leads to the diversity of SCFAs, which indicates that the characteristics of different types of SCFAs in regulating inflammatory response need to be studied more precisely. Most studies have confirmed the role of butyrate in regulating inflammatory responses. It was demonstrated that butyrate supplemented by *Faecalibacterium* ameliorated macrophage infiltration and reduced the levels of Mcpt1, IL-1β, and IL-6 in chronic kidney disease [38]. In alcoholic liver disease, butyrate was also found to significantly increase the plasma IL-10 concentration while decrease the level of plasma TNF-α [39], thus alleviating the progression of liver lesions. Compared with butyrate, acetate and propionate are less reportedly linked with inflammation. However, some evidence showed that there are functional differences among these three SCFAs. It is well known that macrophages are the major cell type of inflammatory mediators involved in inflammatory disease, such as atherosclerosis and rheumatoid arthritis [40,41], and could produce large amounts of TNF-α, IL-6, nitric oxide (NO), and arachidonic acid derivatives once activated. Cox et al. described the inhibitive effect that SCFAs exert, of which butyrate was the most and acetate the less potent, on the production of macrophage chemoattractant protein-1 (MCP-1, also known as CCL2) either in the presence or absence of LPS [42]. Interestingly, all SCFAs dose-dependently inhibited NF-κB activity and propionate dose-dependently suppressed IL-6 mRNA and protein release in inflammation bowel disease. Further studies revealed that propionate and butyrate at 30 mmol/L caused a strong inhibition of immune-related gene expression, whereas acetate was less effective [43]. Interestingly, SCFAs can regulate insulin resistance and reduce the risk of type 2 diabetes (T2DM), which is identified as an important promoter of CVD [44]. It was observed that mice which consumed a high fat diet developed insulin resistance or obesity while butyrate supplements can reverse this progression [45]. Chronic low-grade inflammation is a hallmark of obesity and T2DM and is accompanied by increased levels of pro-inflammatory cytokines, such as TNF-α, IL-1β, IL-2, IL-6, IL-17, and IFN-γ, which further disturb metabolism and aggravate pancreatic β-cell dysfunction and insulin resistance [46]. In addition, disturbance of gut microbiota increase the level of LPS while reducing the SCFAs, which was also considered a pivotal reason for T2DM. Whelan et al. fed mice either a high fat diet, a diet supplemented with LPS, or a control diet. The mice fed LPS developed obesity and T2DM in a similar way as those that were fed a high fat diet [47]. All this evidence supported the anti-inflammation function of SCFAs, while there are feature discrepancies among different type of SCFAs.

Gut microbiota could induce immune cell differentiation and other biological reaction while it may be double-edged. First of all, gut microbiota is necessary for development and maturation of the host immune system. It has been identified that NK cells and gut innate lymphoid cells (ILCs) are intimately associated to the gut microbiota for the reason that immune cells could be developed and programed in the gut by microbiota [48]. For example, microbiota-educated ILCs participate in systemic host defense against infections like pneumonia [49]. Meanwhile, immune cells in the gut also shape the composition of gut microbiota in return. With the depletion of dendritic cells, macrophages, CD4+ T-cells, CD8+ T-cells, and B-cells through monoclonal antibodies and clodronate liposomes, *Enterobacteriaceae*, *Bacteroides acidifaciens*, and *Mucispirillum schaedleri* were highly enriched in the mucosa and lumen of the small intestine [40]. Secondly, innate immune cells can recognize imbalanced gut microbiota directly when the intestinal mucosal barrier is broken. Immune responses are initiated when naive immune cells recognized the antigen associated with Major Histocompatibility Complex class II molecules on the surface of antigen presenting cells [50]. Disrupted gut microbiota lead to the excessive activation of immune cells, and the antigen presenting cells (APC) are the first to act. Dependent on the microbiota, gut dendritic cells, a kind of APC, secrete IL-12 to foster the development of T helper cells type 1 (Th1) [51], while the Toll-like receptors (TLRs) of some specialized tolerogenic dendritic cells induce the formation of regulatory T cells (Treg), which regulate the response towards microbes by secreting IL6, IL-10, and TGF-ꞵ [52]. Nevertheless, a recent study found that APC shows cell specificity in activating immune cells. For example, antigen presentation by cells expressing RORγt, rather than by classical dendritic cells, was required for the induction of Treg cells [53]. This is a complex process and more detailed mechanisms need to be explored.

### 2.3. Disturbance of Gut Microbes Induces Inflammatory Response

Gut microbiota is mainly divided into three categories: symbiotic bacteria, opportunistic pathogenic bacteria, and pathogenic bacteria. The symbiotic bacteria are obligate anaerobes, which are colonized in deep mucosa of the intestinal tract. They are generally stable and beneficial to host health, such as *bifidobacteria, lactobacilli* and other beneficial bacteria, including those suggested to be with probiotic properties [54,55]. While opportunistic pathogenic bacteria like *Enterococcus*, *Enterobacter* perform dual functions [56]. Under normal circumstances, they were well restricted by the symbiotic bacteria and play a beneficial role in health. However, when the symbiotic bacteria are heavily inhibited, opportunistic pathogenic bacteria will proliferate greatly and invade extraintestinal organs through intestinal mucosa. The third category is pathogenic bacteria, such as *salmonella*, pathogenic *E. coli,* etc., which do not settle in the intestinal tract originally because of the resistance of the healthy intestinal tract. However, once excessive pathogenic bacteria invade, insufficient resistance will lead to intestinal infection and systemic inflammatory response [57,58].

The integrity of the intestinal barrier is tightly regulated by gut microbiota and its interplay with immune cells in mucosa. It has been reported that the mucosal barrier was always damaged to some extent in patients with cardiovascular diseases, including myocardial infarction, heart failure, and hypertension [59]. At the same time, pathogenic bacteria immerse into the intestinal mucosa and activate the intestinal sedentary immune cells, causing systemic inflammatory response. Finally, the translocated gut microbiota directly contacts intestinal immune cells in the lamina propria, leading to a soared release of inflammatory factors, aggravating cardiovascular disease [60]. Clinically, the abuse of broad-spectrum antibiotics claims for the gastrointestinal barrier impairment via inhibiting the symbiotic bacteria, allows the infiltration of pathogenic bacteria and its detrimental metabolites into the circulation cycle. Conversely, increasing the number of probiotics is beneficial for gut microbiota function [61]. The Western diet (WD) is a crucial reason for systemic inflammation and cognitive decline in mice. Supplementation of probiotic *Bifidobacteria infantis* in the diet of WD mice could significantly reduce the levels of IL-6, TNF-α, and CD11b in the blood, thereby reducing neuroinflammation. Moreover, these probiotic-derived metabolites could also effectively increase the levels of PSD95 and BDNF in the central nervous system (CNS) through the blood brain barrier (BBB), improving neuroplasticity [62].

There are limited but important evidence for gut microbial disturbances induced inflammatory responses in heart and renal diseases, accompanied by intestinal edema, which could damage the intestinal mucosal barrier, causing the disturbance of the intestinal microbiota [63,64]. Notably, the weakened function of heart and kidney could further deteriorate the microenvironment of the intestine, and the abnormal accumulation of metabolites caused by heart and kidney disease then aggravate the disturbance of gut microbiota in return, leading to more severe local or systemic inflammatory responses [65]. It appears to be a vicious feedback process. Regulating the gut microbiota and maintaining the function of the intestinal barrier have been identified to effectively inhibit systemic inflammatory response in mice with chronic kidney disease, reduce renal fibrosis, and sustain renal function [66]. At present, keeping the stability of gut microbiota by supplementing probiotics or prebiotics has become a common treatment strategy for some chronic diseases, such as hypertension, atherosclerosis, and kidney failure. Probiotics or prebiotics can effectively improve the intestinal microenvironment and greatly control inflammatory bowel disease [67]. Some plant extracts or traditional Chinese medicine preparations, such as plant polysaccharides, could increase the abundance and variety of symbiotic bacteria and inhibit the growth and reproduction of opportunistic pathogenic bacteria. It was demonstrated that supplementation of C. lentillifera polysaccharide (CLP) resulted in a higher α-diversity of gut microbiota while CLP administration had no effect on SCFAs production [68]. At the same time, they stimulate gut microbiota to produce a variety of active substances, including SCFAs, GABA, and 5-HT, which are helpful for strengthening the intestinal barrier, so as to improve the prognosis of a variety of inflammation-related diseases, for example, neurodegenerative diseases [69].

Recent studies showed that metformin (MET) intervention alleviated atherosclerosis which might be closely related to the modification of gut microbiota. The study found that after MET treatment, gut microbiota imbalance was effectively modified, with the increased level of short-chain fatty acids and reduced level of inflammatory cytokines like IL-1β, IL-6, TNF-α, and LPS in the plaque [70]. In conclusion, modulating gut microbiota to reduce the inflammatory response may become an effective intervention for controlling cardiovascular disease.

## 3. Imbalance of Gut Microbes and CVD

### 3.1. Atherosclerosis

Atherosclerosis (AS) is one of the causes of CVD. Lipid deposition and persistent vascular inflammation are considered as two core factors of atherosclerotic plaque progression [71,72]. Previously, few studies linked gut microbiota with atherosclerosis. However, a study based on fecal metagenomics, clinical measurements, and epidemiology has shown that daily diet promoted the formation of atherosclerosis by affecting gut microbiota, while dysfunction of the anti-inflammatory response may be the core process [73].

Currently, diet and gut microbiota are highly correlated with the progression of atherosclerosis, while inflammatory response participated in this process. For example, the increase of Bacteroides fragilis reduced the abundance of *Lactobacilli* and increased the abundance of *Desulfovibrionaceae*, which led to glucose or lipid metabolism dysfunction and aggravation of the inflammatory response [74]. Circulatory low-density lipoprotein content is significantly increased, accompanied with CD36 and F4/80 increasing in plaques [74], which promoted the formation of aortic plaques and the progression of atherosclerosis.

Aging is another key risk factor for the development of atherosclerosis. Studies have shown that aging disrupted the balance of gut microbiota, significantly increasing the abundance of pathogenic microbiota. Moreover, the level of serum LPS increased, accompanied by the decrease of SCFAs level. Metabolomic results showed that components involved in the metabolic pathway of arachidonic acid (AA), such as 20-HETE, PGF2α, arachidonic acid, and LTB4, were significantly increased in aging individuals with aggravated inflammatory response [75]. Therefore, it is highly suggested that gut microbiota and atherosclerosis may be connected through the axis of “gut microbiota—metabolites—local inflammation—atherosclerosis”. Many studies on anti-atherosclerosis drugs have shown that they inhibited the progression of atherosclerosis by regulating gut microbiota [76,77,78]. For example, it was found that peanut skin extract (PSE) decreased the serum TC and LDL-C contents and increased the HDL-C content in atherosclerotic mice, thereby slowing down the atheromatous plaque formation [79]. What is more, gastrodine was found to regulate gut microbial species and abundance, reducing the level of pro-inflammatory cytokines TNF-α and IL-6 and increasing that of anti-inflammatory factors IL-10 [79]. Tongxinluo intervention, a kind of Traditional Chinese Medicine, altered plaque stability by increasing the level of probiotics in the gut, thereby boosting the content of beneficial metabolites, such as trans-ferulic acid, which could inhibit the NLRP3-related inflammatory pathway in plaque and stabilize plaque [80]. In conclusion, there is a close relationship between gut microbiota and atherosclerosis, and modulating gut microbiota may be a new way for the treatment of atherosclerosis.

### 3.2. Myocardial Infarction

Coronary atherosclerosis is the most common cause of myocardial infarction. The relationship between gut microbiota and atherosclerosis has been described above. In addition, gut microbiota is also an important predictor of the occurrence and development of acute myocardial infarction (AMI). The gut microbiota and its related metabolites were detected in 48 normal patients (NCA), 49 patients with AMI, and 93 patients with stable coronary artery disease (sCAD), then it was found that the gut microbiota and metabolites were dramatically changed in patients with AMI. Fourteen types of gut bacteria and 30 kinds of metabolites [81] were involved, including *Acidobacterium*, *Streptococcus*, *Ruminococcus*, *Lactobacillus*, and *Enterococcus*. The disruption of gut microbiota on AMI mainly influences the process of cardiac ischemia-reperfusion injury and cardiac repair after infarction, and inflammation is believed to play a key role in this process.

Although coronary interventional therapy could restore the coronary blood supply in a short time, ischemia/reperfusion (I/R) injury is inevitable. Reactive oxygen species (ROS) is an important risk factor for I/R injury. Gut microbiota activated neutrophils in the blood, forming neutrophil extracellular traps (NETs) and produced a large number of ROS in damaged cardiac cells, which directly led to apoptosis of cardiomyocytes and myocardial microvascular endothelial cells [82]. Notably, the formation of NETs often requires direct contact of neutrophils with microorganisms, suggesting the existence of the impaired intestinal mucosal barrier in AMI.

Gut microbiota regulates the composition of immune cells in the heart after AMI to affect the repair process of the heart. When antibiotics were used to inhibit the gut microbiota of mice after AMI, the drastic reduction of SCFAs was obviously correlated with the decreased proportion of myeloid cells, suggesting that repairment after MI was impaired. While the reconstruction of gut microbiota by fecal transplantation can significantly improve the physiological state and survival time of mice, in which lactic acid bacteria may play an pivotal role [83].

Previous studies suggested the alteration of gut microbiota after AMI, where the stress state and hemodynamic always changed. One study reported that the abundance of *Synergistetes*, *Spirochaetes*, *Lachnospiraceae*, *Syntrophomonadaceae*, *Tissierella*, and *Soehngenia* were increased significantly after AMI [84]. Then it was found that the disorder of gut microbiota decreased the secretion of some metabolites, such as SCFAs. As SCFAs are important in maintaining the physiological state of myocardial function, this impairment may aggravate the prognosis of AMI. Notably, this might be a positive feedback process, as disturbances of gut microbiota also contribute to the progression of coronary atherosclerosis, a major cause of myocardial infarction. In conclusion, although limited studies indicated harmful feedback of “gut microbiota-myocardial cells” in AMI, much more investigation is needed to delineate more details and mechanisms. As a hotspot, supplementing probiotics may be a promising way for the prevention and treatment of myocardial infarction.

### 3.3. Heart Failure

Fecal metagenomic analysis of 53 patients with chronic heart failure and 41 control members showed that gut microbiota composition and metabolic characteristics of patients with chronic heart failure (CHF) were significantly different from those of the control group [85], suggesting that gut microbiota dysfunction was closely related to chronic heart failure. LPS, which was secreted when gut microbiota was disrupted, reduces ZO-1 tight junctions (TJs) in a Toll like receptor 4 (TLR4)-dependent manner and induces an apparent deformation of intestinal epithelial TJs, causing destruction of the integrity of the intestinal barrier [86,87]. In CHF patients with a microbiota disorder of the mucosal epithelium, LPS was then transited through damaged intestinal mucosa into the systemic circulation, directly acting on cardiac myocytes and macrophages to release various proinflammatory cytokines through the stimulation of TLR4, thus exacerbating heart failure [88].

Cardiac remodeling is an early pathological process of heart failure, where myocardial fibrosis is a major initiator. Overload of the heart caused by the weakened systolic function would lead to the disruption of gut microbiota, reducing the bacteria that produce tryptophan and SCFAs in the gut, which increased the infiltration of T cells in the heart, aggravated cardiac fibrosis, and damaged cardiac function [89]. Nevertheless, a few studies have implicated an inflammation lowing effect by appropriately regulating gut microbiota, thus mitigating or reversing the ventricular remodeling [90,91]. At present, some traditional Chinese medicines, such as Qiliqiangxin (QL), were reported to stabilize the gut microbiota after heart failure, inhibiting myocardial fibrosis and cardiac remodeling by reducing the generation of inflammatory factors, such as NLRP3, IL-1B, and TNF-α [92].

Patients with heart failure are deemed to undergo a chronic systemic inflammatory response, and levels of several proinflammatory cytokines in plasma are associated with the severity and prognosis of the disease [93,94]. Gut microbiota disruption and translocation of bacterial products, such as lipopolysaccharide (LPS), into the blood are considered to be main factors of a hyperinflammatory state [95,96]. LPS is one of the strongest proinflammatory mediators [97] and it induces the release of TNF-α, IL-1, and IL6 in the serum of patients with heart failure. On the other hand, LPS directly induces cardiomyocyte damage through Toll-like receptor 4 (TLR4). Studies have shown that the expression of TLR4 is increased in the hearts of patients with advanced heart failure and is highly correlated with cardiac inflammatory injury, while inhibition of TLR4 alleviated the progression of heart failure [98]. Taken together, we hypothesize that the inflammatory response regulated by gut microbiota might be a crucial reason for aggravation of heart failure. Notably, severe heart failure is always accompanied by the dysfunction of intestinal function. Therefore, paying more attention to sustaining the intestinal function might improve the prognosis of patients with heart failure.

### 3.4. Hypertension

It has become a general agreement that there is a tight connection of gut microbiota with blood pressure regulation under the confirmation of many animal models, including spontaneously hypertensive rats (SHR), Ang II–induced hypertensive rats, and Dahl-salt sensitive rats [99,100,101,102,103]. Studies reported that *Bifidobacteria* could increase the activity of nitric oxide synthase (eNOS) while decreasing the activity of serum catalase [104]. Emerging evidence figured out that gut microbiota is involved in neuroinflammation mediated hypertension. For example, neuroinflammation in the hypothalamic paraventricular nucleus (PVN) of SHR could be significantly reduced by FMT from normotensive rats [105]. Interestingly, administration of anti-inflammatory drugs, such as minocycline and CMT-3 in Ang II-induced hypertensive rats, restored gut microbiota communities and attenuated the pathological conditions of the gut wall [106]. Here, we attempt to propose a bidirectional regulation of gut microbiota and hypertension through both humoral and neural regulation pathways, despite limited evidence to date. In the gastrointestinal tract of hypertensive animals, macrophages are the first station connected to gut microbiota influencing the inflammatory response [107]. Disturbed gut microbiota and its dysregulated metabolites stimulate macrophages to release overdose inflammatory factors into the blood, which aggravated the progression of hypertension [108]. Researchers also found important differences in gut microbiota between hypertensive patients and normotensive groups, where lifestyle and dietary habits matter intensively [109]. A high salt diet has long been recognized as a key risk for hypertension, thus unsurprisingly altering the balance of gut microbiota [110]. Despite limited evidence of the mechanisms of how gut microbiota modulates the inflammatory response in hypertension currently, it still poses a novel direction for treatment.

Neuroinflammation contributes to hyper sympathetic activity and is highly related to neurogenic hypertension. Raizada et al. focused on the link between peripheral and central neuroinflammation, autonomic nervous system, and bone marrow, and proposed a complex hypothesis of the brain–gut–bone marrow axis in hypertension [111]. A previous study demonstrated that sympathetic nervous excitement caused by neuroinflammation would mobilize hematopoietic stem cells in the bone marrow and promote the differentiation of inflammatory cells [112], while inflammatory cells would return to the brain and aggravate neuroinflammation. In addition, the activation of the sympathetic nervous system increases the permeability of intestinal mucosa and disrupts the balance of gut microbiota [113]. Disordered gut microbiota released various pathogenic bacteria metabolites into the circulation, which significantly increased central inflammation and promoted the sympathetic nerve excitement [111]. In conclusion, inflammatory response regulation is an important link connecting gut microbiota and hypertension, suggesting an interacted communication between them. In addition, a balanced gut microbiota promotes the absorption and utilization of antihypertensive drugs, which can ameliorate hypertension.

## 4. Therapeutic Strategies Target the Intestinal Microenvironment

Gut microbiota modulation has already been applied in clinical practice. The most common strategy is the dietary therapy, which has been identified to alleviate cardiovascular diseases like atherosclerosis, hypertension, and heart failure. It has been reported that a high-fiber diet increased the abundance of symbiotic bacteria and inhibited the propagation of opportunistic pathogenic bacteria [114], for example, the increased proportion of acetate-producing bacteria effectively reduced blood pressure and alleviated cardiac hypertrophic and fibrosis [115].

Prebiotics are a group of non-digestible carbohydrates that selectively alter microbial composition and activity. Studies have shown that some prebiotics reduce the occurrence of cardiovascular events by regulating various risk factors, such as oxidative stress, renin-angiotensin system overactivity, inflammation, hyperlipidemia, and vascular resistance [116]. Researchers found that administration of *Lactobacilli* reduced toxins originated from disturbed gut microbiota and increased the level of SCFAs, inhibiting the progression of atherosclerosis [117]. Photobiomodulation, a non-thermal light therapy, has been proposed to restore the balance of gut microbiota. It was found to effectively inhibit mitochondrial dysfunction, reactive oxidative stress, inflammation, and gut dysbiosis in chronic kidney diseases, Alzheimer’s disease, and type 2 diabetes mellitus [118,119].

Targeted regulation of intestinal metabolites is also a therapeutic strategy for cardiovascular disease. Gut microbiota originated from metabolite trimethylamine-*N*-oxide (TMAO) is closely related to the pathophysiology of coronary heart disease and is formed when trimethylamine (TMA) is converted by flavin-containing monooxygenases in hepatocytes [120]. Therefore, inhibiting the production of TMA might effectively reduce TMAO production and alleviate the inflammatory response. Flavonoids have the function of treating CHD by inhibiting TMA lyase [121]. Oat fiber feeding was found to prevent the deterioration of atherosclerosis. In addition to promoting atherosclerosis by modifying lipid metabolism, this feeding strategy also blocked the TLR4 signaling pathway, reduced the expression of NF-κB p65, and maintained the integrity of the intestinal mucosal barrier by affecting the intestinal microbiota-derived isobutyry-L-carnitine, valerylcarnitine, 1-methylguanosine, and 2-methylguanosine [122].

Fish oil-derived long-chain monounsaturated fatty acids (LCMUFA) have been shown to ameliorate cardiovascular risk in mice by improving endothelial function [123]. A double-blind, randomized, multicenter study showed that participants of the LCMUFA group exhibited improved endothelial function and lower levels of TMAO, suggesting a significant reduction in coronary heart disease risk [123]. Interestingly, this study found that only a certain specific LCMUFA with a carbon chain length longer than 18 units turned out to be effective. Animal studies were further applied and found that LCMUFAs sustain the balance of the intestinal microenvironment by reducing the proportion of Firmicutes and Bacteroidetes, increasing the abundance of *Akkermansia* in the gut and upregulating SCFAs as well as some glucagon-like substances induced by SCFAs [123]. All these significantly modified the intestinal microenvironment, which reduced the level of inflammatory cytokines in serum and inhibited the progression of atherosclerotic lesions, providing strong evidence that exogenous use of LCMUFAs could improve gut microbiota function, promote SCFAs production, and help prevent cardiovascular disease.

Amino acids have been widely identified as a kind of immune regulated factor [124]. It is demonstrated that branched-chain amino acid (BCAA) supplementation can attenuate atherosclerosis through regulating inflammation, including reducing macrophage infiltration, lowering serum levels of inflammatory factors, and suppressing inflammatory-related signaling pathways [77]. Although there is less research focus on the function of amino acid in regulating gut microbiota, it is a promising way.

**Figure 2:**
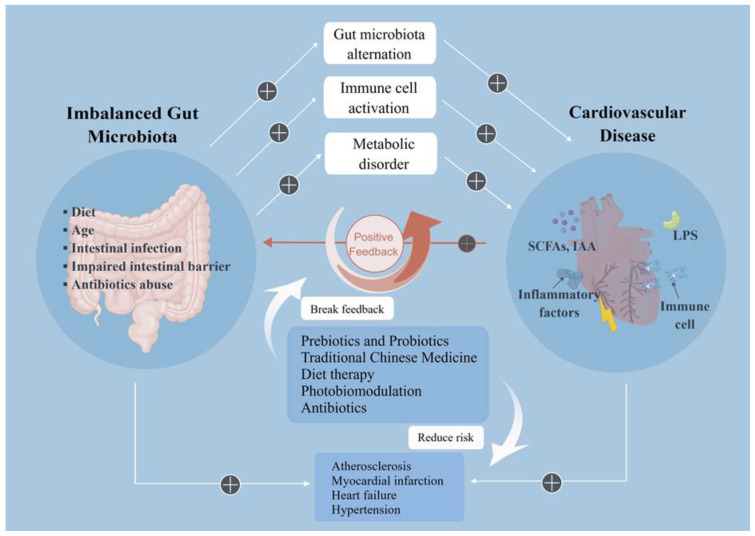
Imbalanced gut microbiota can aggravate cardiovascular disease. Imbalanced gut microbiota caused by dietary habits, age, and antibiotics abuse can aggravate cardiovascular diseases through gut microbiota composition alternation, immune cell activation, and metabolic disorder. The cardiovascular diseases can further promote the disturbance of gut microbiota in return, forming positive feedback. Some treatments like prebiotics and probiotics supplements, TCM, diet therapy, and photobiomodulation can break this feedback and alleviate cardiovascular diseases, such as atherosclerosis, myocardial fraction, heart failure and hypertension. SCFAs: Short-chain fatty acids; IAA: indoleacetic acid; LPS: Lipopolysaccharide. Created with Figdraw.

The close relationship between inflammation and CVD has been widely identified before. Although clinical strategies of gut microbiota for treating CVD remain immature to date, recent findings have demonstrated that stable and balanced gut microbiota uphold protective effects. Therefore, individualized treatment regimens based on gut microbiota may provide new treatment options for patients with CVD (Figure 2).

## 5. Conclusions and Future Perspectives

CVD is one of the most serious public health problems at present. Although clinical treatment strategies are comprehensively complete, the morbidity and mortality of CVD still remain high. With the in-depth study of gut microbiota, its underlying regulating mechanism on the cardiovascular system has been gradually revealed. This review supposes that gut microbiota is involved in the occurrence and development of cardiovascular diseases by regulating local or systemic inflammatory responses. Evidence is notably supporting an active crosstalk between CVD and gut microbiota disturbances. Maintaining the stability of gut microbiota exerts an inhibitory effect on the progression of CVD. Suitable dietary therapy, like probiotics and prebiotics supplementation, can maintain the balance of gut microbiota, which has been proven to effectively reduce the level of inflammatory factors in the blood and improve the prognosis of CVD. What is more, in clinical practice, scholars have used antibiotics to selectively inhibit certain harmful bacteria, so as to alter the composition of gut microbiota and achieve the therapeutic purpose. Now, utilizing gut microbiota-related treatments for CVD has yielded promising results. However, the complex composition of the gut microbiota and the characteristic of each kind of microbiota remain unknown. In addition, there are no relevant guidelines on the clinical use of gut microbiota drugs so far. Therefore, a more accurate analysis for the function of gut microbiota species and its metabolites in CVD urgently needs to be determined.

## Data Availability

Not applicable.
